# Outcome 1 year after ICH: Data from the Tranexamic acid for IntraCerebral Haemorrhage 2 (TICH-2) trial

**DOI:** 10.1177/23969873241265939

**Published:** 2024-07-30

**Authors:** Zhe Kang Law, Chaamanti Sivakumar Menon, Lisa J Woodhouse, Jason Philip Appleton, Rustam Al-Shahi Salman, Thompson Robinson, David Werring, Christine Roffe, Robert A Dineen, Philip Michael Bath, Nikola Sprigg

**Affiliations:** 1 Stroke Medicine, Mental Health & Clinical Neuroscience, University of Nottingham, Nottingham, UK; 2 Department of Medicine, Faculty of Medicine, National University of Malaysia, Kuala Lumpur, Malaysia; 3 Stroke, Nottingham University Hospitals NHS Trust, Nottingham, UK; 4 Centre for Clinical Brain Sciences, University of Edinburgh, Edinburgh, UK; 5 Department of Cardiovascular Sciences and NIHR Leicester Biomedical Research Centre, University of Leicester, Leicester, UK; 6 Institute of Neurology and National Hospital for Neurology and Neurosurgery, University College London, London, UK; 7 Stroke Research, Keele University, Stoke-on-Trent, UK; 8 Radiological Sciences, Mental Health & Clinical Neuroscience, University of Nottingham, Nottingham, UK; 9 NIHR Nottingham Biomedical Research Centre, Nottingham, UK

**Keywords:** Intracerebral haemorrhage, tranexamic acid, randomised controlled trial, survival

## Abstract

**Introduction:**

The Tranexamic acid for IntraCerebral Haemorrhage-2 (TICH-2) trial reported no significant improvement in death and dependency at day 90 despite reductions in haematoma expansion, early neurological deterioration and early death. However, significant recovery after stroke, particularly intracerebral haemorrhage (ICH), may take more than 3 months. Here we report the participant outcomes at 1 year after stroke.

**Patients and methods:**

TICH-2 was a prospective randomised controlled trial that tested the efficacy and safety of tranexamic acid in spontaneous ICH when given within 8 h of onset. Patients with ICH on anticoagulation were excluded. Centralised blinded telephone follow up was performed for patients from the United Kingdom at 1 year. The primary outcome was modified Rankin Scale at 1 year. Secondary outcomes included Barthel index, Telephone Interview Cognitive Status-modified, EuroQoL-5D and Zung Depression Scale. This was a prespecified secondary analysis of the TICH-2 trial.

**Results:**

About 2325 patients were recruited into the trial (age 68.9 ± 13.8 years; 1301 male, 56%). About 1910 participants (82.2%) were eligible for day 365 follow up. 57 patients (3.0%) were lost to follow up. Tranexamic acid did not reduce the risk of poor functional outcome at 1 year (adjusted OR 0.91 95% CI 0.77–1.09; *p* = 0.302). However, Cox proportional hazard analysis revealed significant survival benefit in the tranexamic acid group (adjusted HR 0.83, 95% CI 0.70–0.99; *p* = 0.038).

**Conclusion:**

There was no difference in functional outcome at 1 year after ICH. Tranexamic acid may reduce mortality at 1 year without an increase in severely dependent survivors. But this should be interpreted with caution as this is a result of secondary analysis in a neutral trial.

## Introduction

Acute intracerebral haemorrhage (ICH) accounted for approximately 3.2 million cases of stroke^1^ and 2.89 million deaths in 2019.^2^ In terms of disability-adjusted life years (DALYs), ICH accounted for 4.4% of all stroke-related DALYs in 2019.^2^ ICH carries a high morbidity and mortality rate due to complications such as haematoma expansion, neuroinflammation and brain oedema as well as few successful treatment options. Haematoma expansion occurs in 38% of patients with acute ICH within the first few hours of onset and leads to higher mortality and worse functional outcome.^3,4^ Hence, limiting haematoma expansion with haemostatic therapy is an important therapeutic target. The Tranexamic acid for IntraCerebral Haemorrhage-2 (TICH-2) trial tested the efficacy and safety of tranexamic acid, an anti-fibrinolytic agent, in acute spontaneous ICH, within 8 h of symptom onset.^5^ There was no significant improvement in death and dependency at day 90 despite small but significant reductions in haematoma expansion, early neurological deterioration, early death and serious adverse events.^5^ One possible explanation is the modest reduction in haematoma volume of 1.4 mL may be insufficient to translate into improvement in functional outcome.^5^ The primary outcome of modified Rankin Scale (mRS) was assessed at 90 days, which corresponds to the period of greatest motor recovery after stroke.^6^ However, motor functions of people with stroke due to ICH may continue to improve beyond 3 months^7^. The prevalence of recurrent ICH, ischaemic stroke and other vascular events are cumulative over the years especially in the older ages groups (55–94).^8^ Recent literature supports avoiding early prognostication as it is a poor indicator of long-term recovery.^1,9–13^ Prognostication and morbidity amongst ICH patients with co-morbidities such as diabetes is worse and not always accounted for in trials.^12^ It has also been shown that reduction of haematoma volume in the first month has a positive impact on recovery at 1 year.^12^ Language and cognitive function may also continue to improve up to 2 years after stroke.^14^ In addition, stroke complications are common with approximately 46% of the trial patients experiencing serious adverse events in the first 90 days, thus impairing the recovery process.^5^

Here, we report on outcomes at 1 year after stroke to explore the possibility of a delayed effect of tranexamic acid on improving functional recovery after ICH. We tested the hypothesis that intravenous tranexamic acid given within 8 h of spontaneous ICH reduces death and dependence at 1 year.

## Patients and methods

TICH-2 trial was a prospective multicentre randomised placebo-controlled trial testing the efficacy and safety of intravenous tranexamic acid in patients with acute spontaneous ICH presenting within 8 h of onset. Details of the trial protocol were published.^5,15^ In short, patients with acute ICH were randomised in a double-blinded manner to receive 2 g of intravenous tranexamic acid or matching placebo. Centralised, blinded assessor telephone follow up was performed at 1 year for patients from the United Kingdom only (due to funding/logistical reasons). The primary outcome was modified Rankin Scale at 1 year. Secondary outcomes included death, Barthel index, Telephone Interview Cognitive Status-modified (TICS-M), EuroQoL-5D (EQ-5D) and Zung Depression Scale (ZDS). Ethics approval was gained prior to the commencement of the trial. Analysing these outcomes of participants at 1-year was a pre-specified secondary analysis of the TICH-2 trial.^16^

## Statistical analysis

We followed a pre-specified statistical plan,^16^ and analysed the outcome at 1 year as a shift in the mRS, using ordinal logistic regression, with adjustment for the stratification and minimisation criteria. We tested the assumption of proportional odds using the likelihood ratio test. We also performed sensitivity analyses of the 1-year mRS and as a binary outcome (split at 0–3 vs 4–6). All analyses followed the intention-to-treat principle and kept participants in the groups to which they were allocated by the minimisation algorithm. We analysed prespecified subgroups using ordinal logistic regression, with adjustment for the stratification and minimisation criteria, to assess any variation of the treatment effect on the primary outcome. We performed Cox proportional hazard regression analysis to examine the effect of tranexamic acid on hazard ratio for death. Continuous variables such as EQ-5D Health Utility Score (HUS) and EQ-5D Visual Assessment Score (VAS), Barthel Index, ZDS and TICS-M used multiple variable regression analysis at 1 year. In addition we analysed the difference in mRS, using ordinal logistic regression, and continuous secondary outcomes, using multiple variable regression, between 90 days and 1 year in the two treatment arms separately.

Data are mean (standard deviation, SD), median [interquartile range], odds ratios (OR), hazard ratio (HR) or mean difference (MD) with 95% confidence intervals (CI). Binary logistic regression (Odds Ratio, OR), multiple linear regression (Mean difference, MD) or Cox proportional hazard regression (hazard ratio, HR). Analyses had adjustment for age, sex, time from onset to randomisation, baseline SBP, baseline NIHSS, presence of IVH, history of prior antiplatelets. The analysis was undertaken by LJW using the SAS software 9.4 version.

## Results

**Figure 1.**
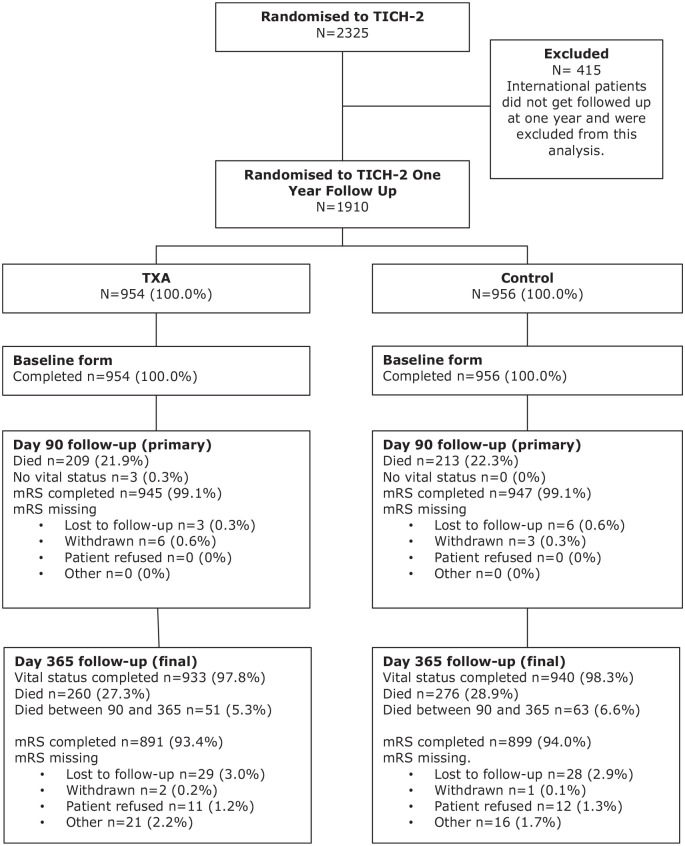
Trial profile.

About 2325 patients were recruited into the TICH-2 trial (age 68.9, (±)13.8 years; 1301 male, 56%). Of these, 1910 participants (82.2%) were eligible for 1 year follow up. At 1 year, 57 patients (3.0%) were lost to follow up, 26 (1.4%) withdrew consent (withdrawal from trial/refused at time of follow-up) and 37 (1.9%) did not complete the follow-up for other reasons, leaving valid 1-year mRS outcome available in 1790 patients (93.7%) (Figure 1). Vital status was available in 1873 patients (Figure 1). The mean age was 69.7 (13.7) and 1041 (54.50%) participants were male. The median time from stroke onset to randomisation was 3.5 h (IQR 2.6–4.8 h). The mean baseline systolic blood pressure was 172.6 mmHg (SD 27.6 mmHg) and diastolic blood pressure was 92.3 mmHg (SD 18.3 mmHg). 543 (56.92%) participants’ haematoma location was supratentorial deep, 325 (34.07%) supratentorial lobar and 61 (6.39%) infratentorial. Mean haematoma volume was 25.3 ml (SD 27.8 ml). There were no baseline differences between the treatment groups followed up till 1-year and those who were followed up till 90 days, apart from country of origin (Table 1).

**Table 1.** Baseline characteristics comparing participant from day 90 follow up and 1 year follow up.

Baseline variable	Day 90 (All patients)	1 year sub-study (all patients, TXA group and Placebo group)		
		All	TXA	Placebo
Participants randomised	2325	1910	954	956
Age (years)*, Mean (SD)	68.9 (13.8)	69.7 (13.7)	69.9 (13.5)	69.5 (13.9)
⩾70 years (%)	1224 (52.65%)	1057 (55.34%)	536 (56.18%)	521 (54.50%)
Sex*, male (%)	1301 (55.96%)	1041 (54.50%)	506 (53.04%)	535 (55.96%)
Onset to randomisation (hours)*, median [IQR]	3.6 [2.6, 5.0]	3.5 [2.6, 4.8]	3.5 [2.6, 4.8]	3.5 [2.5, 4.7]
<3 h (%)	827 (35.57%)	718 (37.59%)	368 (38.57%)	350 (36.61%)
⩽4.5 h (%)	1575 (67.74%)	1360 (71.20%)	672 (70.44%)	688 (71.97%)
History of:				
previous antiplatelet therapy*	611 (26.29%)	518 (27.13%)	266 (27.91%)	252 (26.36%)
statin use prior to admission	622 (26.96%)	556 (29.37%)	287 (30.37%)	269 (28.38%)
previous stroke or Transient Ischaemic Attack	329 (14.30%)	250 (13.24%)	134 (14.26%)	116 (12.24%)
Ischaemic Heart Disease	202 (8.79%)	171 (9.07%)	90 (9.61%)	81 (8.54%)
Pre-stroke mRS, median [IQR]	0.0 [0.0, 1.0]	0.0 [0.0, 1.0]	0.0 [0.0, 1.0]	0.0 [0.0, 1.0]
Glasgow coma scale, Mean (SD)	13.4 (2.1)	13.5 (2.2)	13.4 (2.2)	13.6 (2.1)
NIHSS score*, Mean (SD)	13.0 (7.5)	13.0 (7.7)	13.2 (7.7)	12.9 (7.7)
Systolic blood pressure*, Mean (SD)	172.6 (27.1)	172.6 (27.6)	171.7 (28.1)	173.6 (27.0)
Diastolic blood pressure, Mean (SD)	93.2 (18.1)	92.3 (18.3)	91.7 (18.6)	92.9 (18.0)
Intra-ventricular haemorrhage*, yes (%)	745 (32.04%)	636 (33.30%)	335 (35.12%)	301 (31.49%)
Haematoma location				
Supra-tentorial lobar (%)	738 (31.74%)	643 (33.66%)	325 (34.07%)	318 (33.26%)
Supra-tentorial deep (%)	1371 (58.97%)	1098 (57.49%)	543 (56.92%)	555 (58.05%)
Infra-tentorial (%)	149 (6.41%)	120 (6.28%)	61 (6.39%)	59 (6.17%)
Combination (%)	67 (2.88%)	49 (2.57%)	25 (2.62%)	24 (2.51%)
Intracerebral haematoma volume (ml), Mean (SD)	24.0 (27.2)	25.3 (27.8)	26.0 (28.6)	24.6 (27.0)

Minimisation variables are highlighted with an asterix (*).

**Figure 2.**
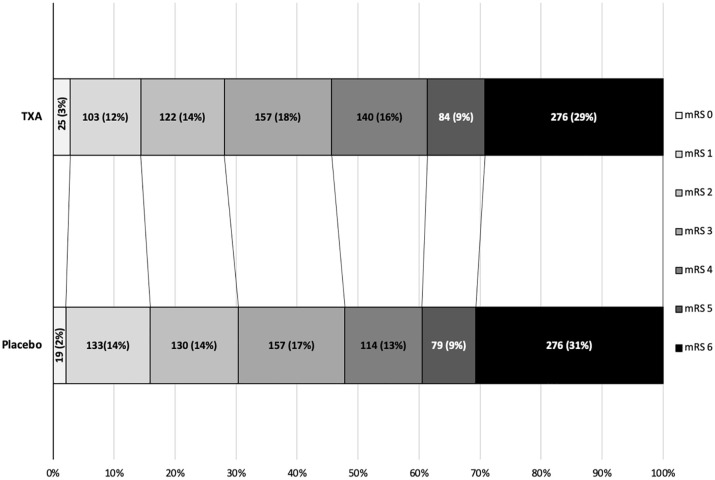
Functional outcome on the modified Rankin Scale (mRS) at 365 days after randomisation. An mRS score of 0 represents no symptoms, 1 represents no disability despite symptoms, 2 represents slight disability but able to look after own affairs, 3 represents moderate disability but able to walk without assistance, 4 represents moderately severe disability (unable to walk or attend to own bodily needs), 5 represents severely disabled (bedridden and requiring constant nursing care), and 6 represents death.mRS = modified Rankin Scale.

**Figure 3.**
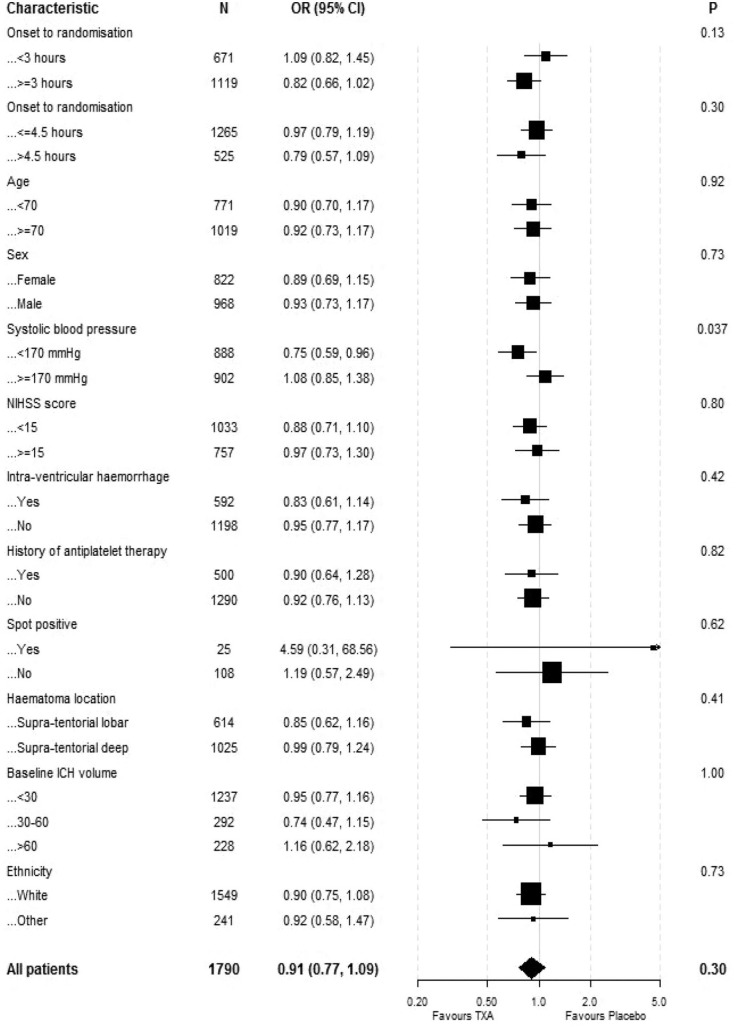
Baseline characteristic differences by subgroups. OR = odds ratio; NIHSS = National Institutes of Health Stroke Scale.

**Figure 4.**
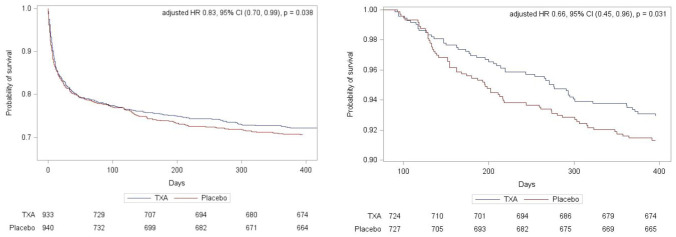
(a) Survival probability following random assignment to tranexamic acid or placebo in 1873 participants recruited in the UK and (b) survival probability in 1415 survivors at day 90 to 1 year.

The primary outcome was assessed in 1790 participants. There were no significant differences in death and dependency (mRS 4–6) in both the ordinal logistic regression analysis at 1 year (adjusted OR 0.91 95% CI 0.77–1.09, *p* = 0.302) (Table 2) or the ordinal shift analysis (Figure 2). We undertook sensitivity analysis of the death and dependency outcome by splitting the mRS scale into dichotomy variables (mRS > 3), however the results were still insignificant (adjusted OR 1.00, 95% CI 0.78–1.29, *p* = 0.97) (Table 2). When we examined the primary outcome within predetermined subgroups (Figure 3), we found that the only significant interaction occurred between mRS and baseline systolic blood pressure (interaction *p* = 0.037). This indicated that participants with a baseline systolic blood pressure of 170 mmHg or lower experienced a beneficial improvement in mRS when treated with tranexamic acid, compared to those with a systolic blood pressure higher than 170 mmHg. There was no heterogeneity of treatment effect by onset of randomisation (Figure 3), whether dichotomised as less than 3 h versus 3 h or longer (interaction *p* = 0.13) or as less than 4.5 h versus 4.5 h or longer (interaction *p* = 0,92); similarly, there was no interaction age <70 versus ≥70 (*p* = 0.92), sex (*p* = 0.73), NIHSS; <15 versus ≥15 (*p* = 0.80), presence of intraventricular haemorrhage (*p* = 0.42), history of antiplatelet therapy (*p* = 0.82), spot sign positive (*p* = 0.62), haematoma location; supratentorial deep versus supratentorial lobar (*p* = 0.41), haematoma volume; <30 ml versus 30–60 ml versus >60 ml (*p* = 0.73) and ethnicity; white versus other (*p* = 0.73) (Figure 3).The majority of deaths occurred before day 90 with only 51 (5%) and 63 (7%) deaths occurring between day 90 and 1 year in the tranexamic acid and placebo group, respectively (Table 2). Cox proportional hazard regression showed significant improvement in survival in the tranexamic acid group (adjusted HR 0.83, 95% CI 0.70–0.99; *p* = 0.037 (Table 2 and Figure 4(a)). Cox proportional hazard regression also showed a statistically significant survival in the tranexamic acid group when analysing the difference in death between day 90 and 1 year (adjusted HR 0.66 (0.45, 0.96 [*p* = 0.031]) (Table 2 and Figure 4(b))).There were no significant differences in quality of life, independency, cognition, and depression at 1 year between groups when assessed using Barthel index, TICS-M, EQ-5D HUS, EQ-5D VAS and ZDS (Table 2).

**Table 2.** Primary and secondary outcomes of the 1 year follow up analysis.

					Adjusted	
	N	All	TXA	Control	OR/MD/HR (95% CI)	*p*
Total number of participants randomised	.	1910	954	956		.
**Primary outcome**	.					.
Total number of participants with outcome (mRS /6)	.	1790	891	899		.
mRS 0	1790	44 (2.46%)	25 (2.81%)	19 (2.11%)	0.91 (0.77, 1.09)	0.3020
mRS 1	1790	227 (12.68%)	103 (11.56%)	124 (13.79%)		.
mRS 2	1790	252 (14.08%)	122 (13.69%)	130 (14.46%)		.
mRS 3	1790	314 (17.54%)	157 (17.62%)	157 (17.46%)		.
mRS 4	1790	254 (14.19%)	140 (15.71%)	114 (12.68%)		.
mRS 5	1790	163 (9.11%)	84 (9.43%)	79 (8.79%)		.
mRS 6, death	1790	536 (29.94%)	260 (29.18%)	276 (30.70%)		.
Sensitivity analysis, day 365	.					.
mRS, median [IQR]	1790	4.0 [2.0, 6.0	4.0 [2.0, 6.0	4.0 [2.0, 6.0		.
mRS, mean (SD)	1790	3.75 (1.87)	3.77 (1.85)	3.74 (1.89)	–0.05 (–0.18, 0.07)	0.4118
mRS > 3, *n* (%)	1790	1267 (70.78%)	641 (71.94%)	626 (69.63%)	1.00 (0.78, 1.29)	0.9733
**Secondary outcomes**	.					.
Day 365	.					.
Death by day 365 (Cox Hazard Ratio), *n* (%)	1873	536 (28.06%)	260 (27.25%)	276 (28.87%)	0.83 (0.70, 0.99)	0.0367
Death between 90 & 365 (Cox Hazard Ratio), *n* (%)	1451	114 (7.86%)	51 (7.04%)	63 (8.67%)	0.66 (0.45, 0.96)	0.031
EQ-5D HUS (/1), mean (SD)	1741	0.33 (0.40)	0.32 (0.40)	0.34 (0.39)	–0.01 (–0.04, 0.03)	0.7405
EQ-VAS (/100), mean (SD)	1101	65.62 (21.65)	65.04 (22.57)	66.19 (20.72)	0.02 (–2.41, 2.46)	0.9848
Barthel Index (/100), mean (SD)	1734	50.55 (46.24)	50.22 (45.94)	50.87 (46.56)	1.59 (–1.66, 4.84)	0.3363
TICS-M (/39), mean (SD)	1069	11.18 (12.89)	11.10 (12.73)	11.25 (13.05)	0.21 (–0.88, 1.30)	0.7097
ZDS (/100), mean (SD)	1116	73.40 (30.12)	73.44 (29.91)	73.35 (30.34)	–0.81 (–3.49, 1.87)	0.5545

Data are *n* (%), mean (standard deviation) or median [interquartile range]. Modified Rankin score (mRS), Telephone Interview Cognitive Status-modified (TICS-M), EuroQoL-5D Health Utility Score (EQ-5D HUS), EuroQoL 5D Visual Assesement Score (EQ-5D VAS) and Zung Depression Scale (ZDS). Analyses are binary logistic regression (Odds Ratio, OR), multiple linear regression (Mean difference, MD) or Cox proportional hazard regression (hazard ratio, HR) with adjustment for age, sex, time from onset to randomisation, baseline SBP, baseline NIHSS, presence of IVH, history of prior antiplatelets.

We further analysed the data with the two treatment arms as seperate subgroups, looking at the outcome differences between day 90 and 1-year (Table 3). We saw no difference in mRS in between day 90 and 1 year in the placebo group or tranexamic group (adjusted OR 1.00, 95% CI 0.85–1.19, *p* = 0.97, adjusted OR 1.10, 95% CI 0.93–1.31, *p* = 0.25 respectively) (Table 3). We also reviewed the continuous secondary outcomes between 90 days and 1 year. Neither placebo nor tranexamic acid group showed any statistically significant difference in the EQ-5D HUS score (MD −0.00, 95% CI −0.04 to −0.03, *p* = 0.76), (MD −0.02, 95% CI −0.05 to 0.01, *p* = 0.16), EQ-5D VAS score (MD 1.93, 95% CI −0.65 to −4.52, *p* = 0.14), (MD 2.44, 95% CI −0.35 to 5.23, *p* = 0.09), or Barthel index (MD −0.36, 95% CI −3.37 to −2.65, *p* = 0.81), (MD −0.03, 95% CI −3.16 to 3.10, *p* = 0.98) (Table 3). However, there was a significant worsening between 90 days and 1 year in the TICS-M and ZDS scores in the placebo group (MD 1.12, 95% CI 0.06 to 2.16, *p* = 0.04 and MD −3.12, 95% CI −5.75- −0.49, p = 0.02 respectively), which was not observed in the tranexamic acid group (MD 0.42, 95% CI −0.66–1.50, p = 0.44, MD −2.26, 95% CI −4.92–0.40, p = 0.10 respectively) (Table 3).

**Table 3.** Primary and secondary outcome differences at day 90 and day 365 shown in all participants, participants that received placebo only and tranexamic acid only.

			Adjusted	
	Day 90	Day 365	OR/MD (95% CI)	*p*
Total number of participants randomised to placebo	940	940		
mRS 0	12 (1.28%)	19 (2.11%)	1.10 (0.93, 1.31)	0.25
mRS 1	95 (10.11%)	124 (13.79%)		
mRS 2	152 (16.17%)	130 (14.46%)		
mRS 3	145 (15.43%)	157 (17.46%)		
mRS 4	176 (18.72%)	114 (12.68%)		
mRS 5	147 (15.64%)	79 (8.79%)		
mRS 6, death	213 (22.66%)	276 (30.70%)		
mRS, median [IQR] {range}	4.0 [2.0, 5.0] {0, 6}	4.0 [2.0, 6.0] {0, 6}		
EQ-5D HUS (/1), mean (SD)	0.32 (0.39)	0.34 (0.39)	–0.02 (–0.05, 0.01)	0.16
EQ-VAS (/100), mean (SD)	46.58 (33.07)	43.88 (35.90)	1.93 (–0.65, 4.52)	0.14
Barthel Index (/100), mean (SD)	51.33 (44.09)	50.87 (46.56)	–0.36 (–3.37, 2.65)	0.81
TICS-M (/39), mean (SD)	12.72 (12.88)	11.25 (13.05)	1.12 (0.06, 2.19)	0.04
ZDS (/100), mean (SD)	69.58 (30.28)	73.35 (30.34)	–3.12 (–5.75, –0.49)	0.02
Total number of participants randomised to tranexamic acid	933	933		
mRS 0	17 (1.82%)	25 (2.81%)	1.00 (0.85, 1.19)	0.97
mRS 1	87 (9.32%)	103 (11.56%)		
mRS 2	165 (17.68%)	122 (13.69%)		
mRS 3	133 (14.26%)	157 (17.62%)		
mRS 4	171 (18.33%)	140 (15.71%)		
mRS 5	151 (16.18%)	84 (9.43%)		
mRS 6, death	209 (22.40%)	260 (29.18%)		
mRS, median [IQR] {range}	4.0 [2.0, 5.0] {0, 6}	4.0 [2.0, 6.0] {0, 6}		
EQ-5D HUS (/1), mean (SD)	0.32 (0.39)	0.32 (0.40)	–0.00 (–0.04, 0.03)	0.76
EQ-VAS (/100), mean (SD)	47.14 (33.73)	43.74 (36.04)	2.44 (–0.35, 5.23)	0.09
Barthel Index (/100), mean (SD)	51.09 (44.44)	50.22 (45.94)	–0.03 (–3.16, 3.10)	0.98
TICS-M (/39), mean (SD)	12.46 (12.42)	11.10 (12.73)	0.42 (–0.66, 1.50)	0.44
ZDS (/100), mean (SD)	68.91 (29.75)	73.44 (29.91)	–2.26 (–4.92, 0.40)	0.10

Data are n(%), mean (standard deviation) or median [interquartile range]. Modified Rankin score (mRS), Telephone Interview Cognitive Status-modified (TICS-M), EuroQoL-5D Health Utility Score (EQ-5D HUS), EuroQoL 5D Visual Assesement Score (EQ-5D VAS) and Zung Depression Scale (ZDS). Analyses are binary logistic regression (Odds Ratio, OR), multiple linear regression (Mean difference, MD) with adjustment for age, sex, time from onset to randomisation, baseline SBP, baseline NIHSS, presence of IVH, history of prior antiplatelets and treatment allocation.

## Discussion

In this pre-specified sub-study, tranexamic acid did not improve functional outcome compared to placebo 1 year after ICH. There was no difference in other secondary outcomes between treatment groups at 1 year.

Cox proportional hazard regression showed significant improvement in survival in the tranexamic acid group at 1-year and the benefit appeared to be cumulative. However, this was a secondary analysis, in a neutral trial, therefore this result may be due to chance. Contrary to the reduction in early death (<7 days) in the primary TICH-2 analysis, the survival curves converge till day 100 before diverging. Possible physiological reasons for a delayed effect on mortality could be the reduced haematoma volume or fewer serious adverse events (SAEs) up until 90 days post stroke in the tranexamic acid group which may have improved the recovery trajectory. However, we cannot draw conclusions on this as SAEs or fatal SAEs were not collected past 90 day and the primary TICH-2 analysis on survival at 90 days was conducted on a larger cohort.

The majority of survivors continued to be dependent at 1-year after ICH, highlighting the burden of disability after ICH. There was no increase in the proportion of survivors with increased disability, suggesting that if there is a survival benefit with tranexamic acid, this is not at the cost of significant disability. The proportion of patients who were dead or dependent is similar to previous literature.^17^ The latest literature supports the possible change in recovery trajectory seen in ICH patients when they are followed up for a longer time-period.^12^ This highlights the need for extended data collection in future studies to further enhance our understanding of sustained intervention effects and improve the reliability of outcome assessments.

In addition, tranexamic acid did not improve cognition, depression, independency or quality of life at 1 year. While the placebo arm showed deterioration in cognition and depression scores at two separate time points (90 days and 1 year), this was not observed in the tranexamic acid group. However, this finding must be interpreted with caution, as it is hypothesis-generating and not statistically significant as a secondary outcome in a trial where the primary outcome was neutral. Studies have shown the increased incidence of dementia and the faster progression of cognitive impairment following an ICH.^18,19^ Previous literature has also noted that the presence of depression at 1 year post ICH worsens their disability despite the initial severity of their haemorrhage.^20^ There is a lack of studies exploring the effect of treatments on outcomes such as cognition and mood especially on long-term recovery and it is important to consider this in future studies.^11,21^

Although the loss to follow up rate increased by nearly 10-fold from day 90 to 1 year follow up, there was still a low number (3%) of patients that withdrew or were lost to follow up. This supports the feasibility of future trials having longer follow up. To our knowledge, this study is one of the largest randomised controlled trials of ICH patients with a 1-year follow up. A limitation of the study is that fatal SAEs and safety outcomes were only reported till day 90, and only up to 7 days for other SAEs. This sub-study was only able to include participants recruited from the UK and not the all the TICH-2 participants were included. Furthermore, this study is a secondary analysis of the TICH-2 trial, and death was not the primary outcome of this study. Therefore, it is possible that the difference seen on the survival analysis is due to chance and we cannot draw robust conclusions or further explore the potential reasons for difference in survival after 90 days.

## Conclusions

In summary, tranexamic acid had no significant benefit on functional outcome 1 year after ICH. This study reassures us that tranexamic acid does not prolong life at the cost of severe disability. The results highlight that many patients with ICH have poor outcomes, with significant mortality, disability, and reduced cognition and quality of life at 1 year. Better treatments are urgently needed for ICH. Whether limiting haematoma growth with early haemostatic therapy (<4.5 h) (as is being tested in current ongoing trials TICH-3 (ISRCTN97695350), INTRINSIC (NCT05836831), THE-ICH trial (ChiCTR1900027065)) leads to clinical benefit or improves outcome remains uncertain.
